# Macrophage-Derived Extracellular Vesicle Promotes Hair Growth

**DOI:** 10.3390/cells9040856

**Published:** 2020-04-01

**Authors:** Ramya Lakshmi Rajendran, Prakash Gangadaran, Chang Hoon Seo, Mi Hee Kwack, Ji Min Oh, Ho Won Lee, Arunnehru Gopal, Young Kwan Sung, Shin Young Jeong, Sang-Woo Lee, Jaetae Lee, Byeong-Cheol Ahn

**Affiliations:** 1 Department of Nuclear Medicine, School of Medicine, Kyungpook National University, Daegu-41944, Korea; ramyag@knu.ac.kr (R.L.R.); prakashg@knu.ac.kr (P.G.); ojm0366@naver.com (J.M.O.); howonlee1234@gmail.com (H.W.L.); arunnehru.gopal@gmail.com (A.G.); syjeong@knu.ac.kr (S.Y.J.); swleenm@knu.ac.kr (S.-W.L.); jaetae@knu.ac.kr (J.L.); 2 Department of Nuclear Medicine, Kyungpook National University Hospital, Daegu-41944, Korea; 3 BK21 Plus KNU Biomedical Convergence Program, Department of Biomedical Science, School of Medicine, Kyungpook National University, Daegu-41944, Korea; ysung@knu.ac.kr; 4 New Drug Development Center, Daegu-Gyeongbuk Medical Innovation Foundation (DGMIF), Daegu-41061, Korea; schch84@dgmif.re.kr; 5 Department of Immunology, School of Medicine, Kyungpook National University, Daegu-41944, Korea; go3004@knu.ac.kr

**Keywords:** macrophage, extracellular vesicles, Wnt, β-catenin signaling, hair follicle, dermal papilla

## Abstract

Hair loss is a common medical problem affecting both males and females. Dermal papilla (DP) cells are the ultimate reservoir of cells with the potential of hair regeneration in hair loss patients. Here, we analyzed the role of macrophage-derived Wnts (3a and 7b) and macrophage extracellular vesicles (MAC-EVs) in promoting hair growth. We studied the proliferation, migration, and expression of growth factors of human-DP cells in the presence or absence of MAC-EVs. Additionally, we tested the effect of MAC-EV treatment on hair growth in a mouse model and human hair follicles. Data from western blot and flow cytometry showed that MAC-EVs were enriched with Wnt3a and Wnt7b, and more than 95% were associated with their membrane. The results suggest that Wnt proteins in MAC-EVs activate the Wnt/β-catenin signaling pathways, which leads to activation of transcription factors (*Axin2 and Lef1*). The MAC-EVs significantly enhanced the proliferation, migration, and levels of hair-inductive markers of DP cells. Additionally, MAC-EVs phosphorylated AKT and increased the levels of the survival protein Bcl-2. The DP cells treated with MAC-EVs showed increased expression of vascular endothelial growth factor (VEGF) and keratinocyte growth factor (KGF). Treatment of Balb/c mice with MAC-EVs promoted hair follicle (HF) growth in vivo and also increased hair shaft size in a short period in human HFs. Our findings suggest that MAC-EV treatment could be clinically used as a promising novel anagen inducer in the treatment of hair loss.

## 1. Introduction

Hair loss is a common disorder resulting from genetic, hormonal, traumatic, and iatrogenic events. It can cause significant psychological stress and adversely affects self-confidence [1,2]. Hair follicle (HF) is a self-renewing structure that undergoes a cycle of phases: anagen, catagen, and telogen. The anagen stage is a rapid proliferation of follicular epithelial cells known as matrix cells in the hair bulb, which then differentiate to make the hair fiber and follicular root sheath cells. Bulb matrix cells are under the control of specialized mesenchymal cells in the dermal papilla. The catagen stage is an apoptosis leading to regression of the lower two thirds of the follicle, preserving the stem cell region, and telogen is a relatively inactive period between the growth phases [2]. HF is composed of mutually dependent layers of the dermis and epidermis [3]. As the complexity of the mechanism involved in HF growth is not well understood, there is an underlining need to decipher it. The development of the fully functional HF renewal system is complex, and it is believed to involve rearrangement of stem cells and their niches [4]. The primary treatment options for hair loss are drugs and transplantation of autologous HF. However, HF donors are few and usually thousands of hair follicles are needed for a pleasant appearance, and drugs (finasteride and minoxidil) only slow down the process of hair fall for a short time [2,5]. Therefore, novel strategies with hair growth capability are needed for hair loss treatment.

Extracellular vesicles (EVs) are natural nano-membranous vesicles released by most cells in extracellular space and culture media [6,7,8,9,10], consisting of proteins, lipids, and nucleic acids [11,12,13]. Studies have shown that EVs play a crucial role in cell–cell communication [12,14]. Numerous recent studies have used immune-cell-derived EVs for therapeutic strategies in many diseases [14,15,16,17]. Few studies have described the role of macrophages in hair growth and regeneration [18,19,20]. A recent study identified the novel involvement of perifollicular macrophages in the activation of skin epithelial stem cells, as an additional signal that regulates HF growth [18]. Another study showed that macrophages induce HF growth through tumor necrosis factor (TNF)-induced AKT/β-catenin signaling in Leucine-rich G-protein-coupled receptor 5 (Lgr5)^+^ HF stem cells [20]. A recent study clearly showed that human perifollicular macrophages maintain the anagen stage in humans by activating DP cells by secreting Wnt proteins [21]. However, the effect of macrophage-derived EVs (MAC-EVs) on the activation of dermal papilla (DP) cells and hair growth is unknown. In this study, we, for the first time, investigated the effect of EVs extracted from the supernatant of cultured macrophages on DP cell activation, to understand the underlining molecular mechanism of HF generation in vitro and in vivo using a mouse model.

## 2. Materials and Methods

### 2.1. Cell Culture

Murine Raw 264.7 cell line (ATCC) was cultured in Dulbecco’s Modified Eagle Media (DMEM)-high glucose (HyClone, Logan, UT, USA), supplemented with 10% EV-depleted fetal bovine serum (FBS) (Hyclone) (18 h, at 120,000× *g* and 4 °C) and 1% penicillin-streptomycin (Gibco, Carlsbad, CA, USA), at 37 °C, with 5% CO_2_.

### 2.2. Isolation of Dermal Papilla Cells

The anagen-phase HFs were obtained from scalp skin after informed consent was obtained from the patients. The study was approved by the Medical Ethical Committee of Kyungpook National University and Hospital (Daegu, Republic of Korea) and was conducted in accordance to principles and guidelines of the Declaration of Helsinki. DP cells were isolated from the bulbs of dissected hair follicles, transferred to tissue culture dishes coated with bovine type I collagen, and cultured in DMEM low-glucose (HyClone, Logan, UT, USA) supplemented with 1 × Antibiotic-Antimycotic, 1 ng/mL bovine fibroblast growth factor, and 20% heat inactivated FBS at 37 °C. The explants were cultured for 7 days, and the medium was changed every 3 days. The isolated DP cells were then plated in 100 mm culture dishes containing DMEM low-glucose, supplemented with 10% heat-inactivated FBS. The cells were sub-cultured according to the percentage of confluence, and cell passage number 2 was used in this study [2].

### 2.3. Isolation of Extracellular Vesicles and Condition Media for Macrophages

When the cells were about 80% confluent, extracellular vesicles were extracted from the culture media of macrophages using ultracentrifugation, as described previously with modification [7]. Briefly, the medium was centrifuged at 1500× *g* for 10 min, at 2000× *g* for 20 min, and then at 10,000× *g* for 30 min, at 4 °C, to remove the unwanted cells and debris. Next, the supernatant was filtered through a 0.45 µm pore size filter. A small portion of the medium was collected, called EV-media (EV-M; media containing EVs), and stored at −80 ℃ until experimental use. This medium was then ultra-centrifuged at 100,000× *g* for 60 min, and the supernatant was collected, called EV-depleted media (EV-DM; media containing no EVs), and stored at −80 ℃. The EV pellets were washed with phosphate-buffer saline (PBS) by ultracentrifugation, as stated above, reconstituted with 50–100 µL PBS, and stored at −80 ℃. The ultracentrifugation was performed using a SW28 rotor, and ultra-clear tubes of optima TML-100 XP ultracentrifuge (Beckman Coulter, GA, USA). The EV concentrations were measured by Pierce Bicinchonic Acid Protein Assay Kit (Thermo Fisher Scientific, MA, USA) and represented as its total protein concentration (per mL) in this study.

### 2.4. Western Blot Analysis

Western blot analysis was performed as described in a previous study [7]. Whole cells and EV-lysates were prepared in Sodium Dodecyl Sulfate (SDS) lysis buffer (62.5 mM Tris, pH 6.8, 2% SDS, 0.1% β-mercaptoethanol, 10% glycerol, and protease inhibitor cocktail (Sigma, MO, USA). Equal amounts of protein were loaded and separated using 10% SDS- polyacrylamide gel electrophoresis. The proteins were transferred to polyvinylidene difluoride (PVDF) membranes (Millipore, MA, USA), probed first with the primary antibody, and then with the secondary antibody conjugated with horseradish peroxidase (see Supplementary Table S1 for details). The signals were detected using enhanced chemiluminescence (GE Healthcare, IL, USA) according to the manufacturer’s instructions. Blot images were cropped and prepared using Picasa3 (version 3.9.1.4.1) (Google, CA, USA) and/or PowerPoint program (Microsoft, WA, USA) (contrast was adjusted, if necessary, for better visualization). Band intensity was measured by GelQuant.NET software (Version 1.8.2) (BiochemLabSolutions.com, CA, USA).

### 2.5. Transmission Electron Microscopy (TEM)

The MAC-EVs pellets were resuspended in 100 µL of 2% paraformaldehyde. Next, 5 µL EVs pellets were attached to the Formvar-carbon coated with EM grids, and covered with protective material like aluminum foil for 20 min to avoid any damage/dryness to the sample. About 100 µL of PBS was added on a sheet of parafilm and grids were transferred on to the drops of PBS, using sterile forceps for washing. Next, it was transferred to 50 µL of 1% of glutaraldehyde and incubated at 25–30 ℃ for 5 min, and then washed with distilled water for 2 min. Samples were stained using 2% uranyl acetate. These steps were repeated 7 more times, and samples were allowed to completely dry before observing under an HT 7700 transmission electron microscope (Hitachi, Tokyo, Japan) to view the size of the EVs [6].

### 2.6. Nanoparticle Tracking Analysis (NTA)

The measurement of size of MAC-EVs was performed by Nano Sight LM 10 (Malvern, Worcestershire, UK) according to the instructions provided. The sample was diluted 1000-folds in milli-Q water, a sterile syringe was used to inject the sample into the chamber, and measurements were done three times, as described previously, and analyzed [6].

### 2.7. Flow Cytometry

MAC-EVs were made to attach to 4 μm aldehyde/sulfate latex beads (Invitrogen, CA, USA) by mixing 5 μg of MAC-EVs with a 10 μL volume of beads for 15 min. PBS was added to a final volume of 1 mL and the mixture was incubated in a rotary shaker for 2 h at room temperature. The reaction was stopped by adding 100 mM glycine and 2% BSA dissolved in PBS, and the mixture was again incubated in a rotary shaker for 30 min at room temperature. For every 10 μL of the MAC-EV-bound beads, 10 μL of Wnt3a and Wnt7b antibody mixture was incubated overnight at 4 °C, after which the mixture was centrifuged at 15,000× *g* for 2 min. The resulting supernatant was discarded. Alexa Fluor Fluorescein isothiocyanate (FITC) anti-rabbit antibody with 2% BSA dissolved in PBS was added to the mixture, which was then incubated at 37 °C for 60 min. Samples were diluted to 1 mL with 2% BSA in PBS and centrifuged for 2 min at 15,000× *g*. The supernatant was discarded, and the beads were resuspended in 1 mL PBS for flow cytometric analysis performed using a BD FACS Aria III instrument (BD Biosciences, NJ, USA).

### 2.8. EV Interaction and Internalization Assay

The interaction and internalization of MAC-EVs were analyzed with confocal microscopy [14]. The EVs were labeled with lipophilic dye (DiD) by incubating for 20 min at room temperature and washing with PBS by ultracentrifugation, as mentioned above. The DiD-labelled EVs (10 and 20 µg) were incubated with human DP cells for 1 h at 37 °C, before methanol fixation. Antifade agent was used to mount the coverslips (Vector Laboratories, CA, USA). The uptake of EV was observed by LSM 800 Laser scanning microscopy (Carl Zeiss, Baden-Württemberg, Germany).

### 2.9. In Vitro Cell Proliferation Assay

The DP cells (5000 cells/well) were seeded in 96-well plates and incubated with PBS, EV-DM (100%), EV-M (100%), and MAC-EVs (5, 10, 15, and 20 µg) for 24 h at 37 °C. The proliferation was measured at 450 nm using a Cell Counting Kit-8 (CCK-8) (Dojindo Molecular Technologies, Kyushu, Japan) according to the manufacturer’s protocol.

### 2.10. In Vitro Cell Migration Assay

Wound-healing migration assay was performed as previously described [2]. The cells were plated in 6-well plates (about 5000 per well) and incubated at 37 ℃, until the cells reached 95% confluence. A scratch was made using a 10 µL pipette tip and washed with PBS to avoid the debris. Next, PBS, EV-DM (100%), EV-M (100%), and MAC-EVs (10 and 20 µg) were added in media of two concentrations containing 20 µg/mL mitomycin C, to inhibit cell proliferation, and imaged using a phase contrast microscope at 0, 12, and 24 h.

### 2.11. RNA Extraction and Reverse Transcriptase Polymerase chain reaction (RT-PCR)

The DP cells were treated with PBS, MAC-EVs (0.1, 0.5, 1 µg/mL), EV-DM (100%), and EV-M (100%) for 24 h. RNA was isolated using TRIZOL reagent (Bioscience technology, South Korea) and cDNA was prepared using a high-capacity cDNA Synthesis Kit (ABI, CA, USA) according to the manufacturer’s protocol. The RT-PCR were performed as previously described [2,22] (Table S2).

### 2.12. Real-Time Polymerase Chain Reaction (Real-Time PCR)

All reactions included SsoAdvanced^TM^ Universal SYBR Green Supermix (Bio-Rad, CA, USA), 50 ng of cDNA, and 10 μM primers. Amplification was performed under the following cycling conditions: 95 °C for 10 min, followed by 40 cycles of 95 °C for 15 s, and 60 °C for 60 s in CFX96 touch-real-time PCR (Bio-Rad, CA, USA). Differences between samples and controls were calculated using the real-time PCR analysis software (Bio-Rad, CA, USA). See Supplementary Table S3 for primer details.

### 2.13. Immunofluorescence (IF) Assay

Immunofluorescence (IF) was performed as described previously [23]. Briefly, macrophages cells (*n* = 10,000) were seeded in 8-well chamber slides and incubated overnight. Cells were fixed with chilled 100% methanol at −20 °C, blocked with 5% BSA for 1 h, and sequentially incubated for 1 h with indicated primary antibody (CD68, Wnt3a, and Wnt7b) in 5% BSA (Supplementary Table S1) and Alexa Fluor-555-anti-rabbit or anti-goat rabbit-FITC secondary antibody. Coverslips were then mounted using VECTASHIELD Antifade mounting medium (Vactor laboratories, CA, USA) and sealed. Confocal images were obtained using an LSM 800 laser scanning microscope (Zeiss, Baden-Württemberg, Germany).

### 2.14. In Vivo Experiments and HFs Weight Measurement

Male 5.5 week Balb/c mice were purchased from Hamamatsu (Shizuoka, Japan). The animals were maintained, and experiments were performed as per the guidelines for care according to the use of laboratory animals of Kyungpook National University. Two days before starting the experiment, the hair from dorsal skin was removed with an electric shaver and the animals were divided into four groups, control (100 µL PBS; *n* = 7), treatment 1 (100 µg MAC-EVs; *n* = 7), treatment 2 (50 µg MAC-EVs; *n* = 7), and positive control (3% Minoxidil; *n* = 7) groups. Animals (7 weeks) were injected intradermally (3 times weekly) for both treatments and control. For the positive control, minoxidil was applied topically (3 times weekly) for 4 weeks as described [2]. After 28 days, the mice were sacrificed by cervical dislocation. Dorsal skin, with hair and without hair, of all the mice from each group was cut (1 cm^2^ area), and excessive fats or other components were removed and weighed on an analytical balance. After measurements, the hair weight was calculated [24].

### 2.15. Histological Analysis

The dorsal skin of all the groups was fixed in 10% formalin and assessed with hematoxylin and eosin (H&E) staining [25]. The number of HFs were counted. Thickness of dermis (cross-section) was taken from the visible microscopic field (3 fields) with at-least 7 measurements, using ZEN lite 2.3 (Carl Zeiss, Baden-Württemberg, Germany).

### 2.16. Human Hair Shaft Elongation

Human HFs were isolated and cultured as previously mentioned [26]. Briefly, biopsy specimens were obtained from the occipital scalps of male patients with androgenic alopecia during hair transplantation with written informed consent from the patients. The Medical Ethical Committee of the Kyungpook National University Hospital (Daegu, Korea) approved all the described studies. The HFs from non-balding scalps were isolated and the subcutaneous fat portions of scalp skin including the lower hair follicles were dissected from the epidermis and dermis. Then, hair follicles were isolated under a binocular microscope by using forceps and maintained in Williams E media without phenol red (Sigma, MI, USA) at 37 °C in a humidified atmosphere of 95% O_2_ and 5% CO_2_, using HFs from 5 individuals for control-PBS, MAC-EVs and HFs from 3 individuals for EV-DM and EV-M. Subsequently, HFs were treated with PBS, MAC-EVs (0.1, 0.5, 1µg/mL), EV-DM (100%), and EV-M (100%). The hair shaft elongation was subsequently measured at days 3 and 6.

### 2.17. Statistical Analysis

All data were expressed as the means ± standard deviation (SD). Two groups of data were statistically analyzed by Student’s *t*-test in MS Office Excel sheet (Microsoft, WA, USA) or GraphPad Prism7 software version 7.04 (GraphPad Software, Inc., CA, USA). A *p*-value less than 0.05 was considered statistically significant.

## 3. Results

### 3.1. Characterization of MAC-EVs

**Figure 1:**
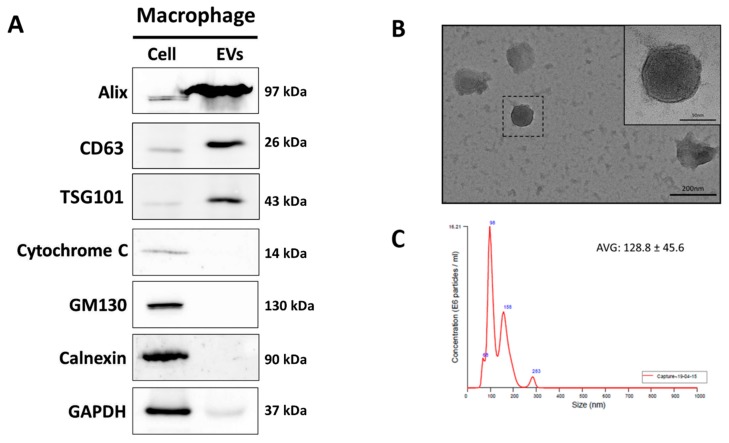
Successful isolation and characterization of MAC-EVs. (**A**) Western blot analysis of cell compartment markers of macrophages (cell) and MAC-EVs (EV). (**B**) Morphology of MAC-EVs confirmed by transmission electron microscopy (scale bar: 50 and 200 nm). (**C**) Size of MAC-EVs determined by NTA (average diameter: 128.8 ± 45.6 nm). EV: Extracellular Vesicles.

Macrophages were confirmed by staining of a macrophage marker CD68 (also known as Gp110 or macrosialin, is a type I transmembrane glycoprotein; Supplementary Figure S1). MAC-EVs were isolated using an ultracentrifuge, as shown in Supplementary Figure S2. To investigate the integrity of MAC-EVs isolation, we examined well-characterized EV markers, both positive and negative, using western blot. As expected, the positive markers, Alix and TSG101 (a cytoplasmic protein), and CD63 (a membrane protein), were enriched in EVs. The negative markers, such as cytochrome C, GM130, and calnexin (markers for mitochondria, Golgi apparatus, and endoplasmic reticulum, respectively) were detected on the cell but not in the EVs. This confirms that the EVs obtained were pure and not contaminated with cells and apoptotic bodies (Figure 1A). The morphology of EVs was evaluated with Transmission electron microscope (TEM) (Figure 1B). The size of the EVs was analyzed by nanoparticle tracking analysis (NTA) and the average size was 128.8 ± 45.6 nm (Figure 1C). These results thus confirmed that MAC-EVs were successfully isolated.

### 3.2. Identification of Hair Growth Inducing Wnt Proteins in EVs and Its Membrane

**Figure 2:**
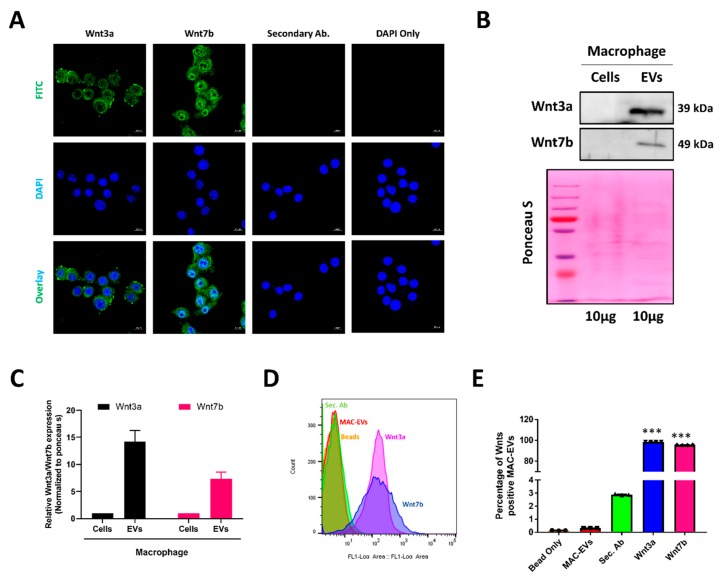
Identification of Wnt3a/Wnt7b protein in MAC-EVs and its association with their membranes. (**A**) Immunofluorescent assay of Wnt3a/Wnt7b in macrophages (scale bar: 10 µm). (**B**) Western blot analysis of Wnt3a and Wnt7b in macrophages (Cell) and MAC-EVs (EV). Ponceau S staining used as loading control. (**C**) Quantification (fold) of Wnt3a and Wnt7b in macrophages (Cell) and MAC-EVs (EV) (*n* = 3). (**D**) Representative flow cytometry count graph of beads only, Control (MAC-EVs), Control (Secondary FITC Antibody), Wnt3a and Wnt7b. (**E**) Percentage of Wnt3a and Wnt7b positive MAC-EVs (*n* = 4) from total population. Means ± standard deviation (SD) of experiments are shown. ***p < 0.001. Student’s t-test was used for comparison. EV: Extracellular Vesicles.

To identify factors responsible for MAC-EV-induced beneficial properties for hair inductivity, we examined the vital proteins such as Wnt3a and Wnt7b [27,28,29] in macrophages and MAC-EVs. First, we confirmed the abundant presence of Wnt3a and Wnt7b in macrophages by immunofluorescent assay (Figure 2A). Western blot analysis revealed that Wnt3a (14.2 ± 2.1-fold) and Wnt7b (7.4 ± 1.2-fold) proteins were enriched in MAC-EVs, when compared to macrophages (Figure 2B, C). The Wnt receptors (Frizzled and LRP5/6) were present on the cell membrane; thus, to activate Wnt/β-catenin signaling, Wnt3a/Wnt7b should also be present at the membrane of MAC-EVs. We performed flow cytometry to confirm the location of Wnt3a/Wnt7b in MAC-EVs and observed that >98% and 95% of MAC-EVs were positive for Wnt3a and Wnt7b, respectively (*p* < 0.001). No signals were detected for beads and MAC-EV control. Low or negligible (<3%) signals were detected for the secondary antibody control (Figure 2D,E), which confirmed that Wnt3a/Wnt7b was present on the EV membrane. Taken together, these results demonstrated that these two Wnt proteins were enriched in MAC-EVs and located on their membranes.

### 3.3. The MAC-EVs Attach to DP Cell Membrane and are Internalized

**Figure 3:**
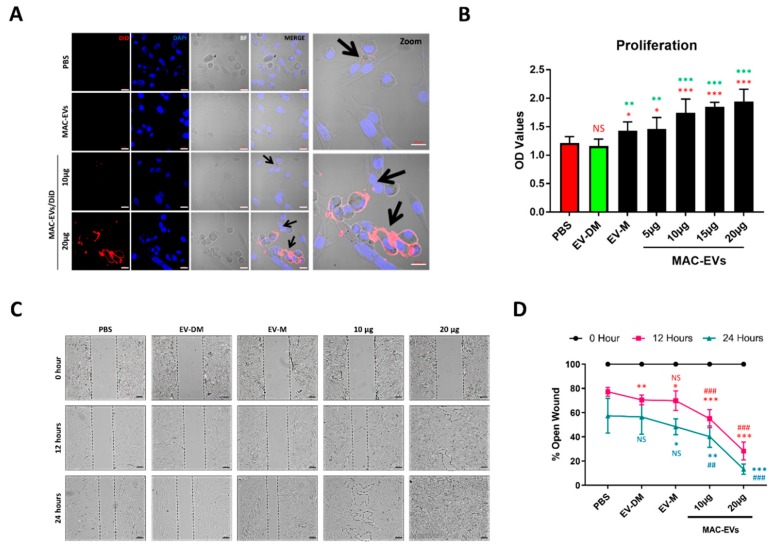
Interaction of MAC-EVs with DP cells leads to cell migration and proliferation. (**A**) DP cells incubated with either PBS, non-labelled MAC-EVs, and DiD-labelled MAC-EVs (MAC-EVs/DiD), arrows indicate the internalization, scale bar: 20 µm. (**B**) Proliferation of DP cells shown in dot blots obtained by CCK8 assay after 24 h of treatment with MAC-EVs (0 to 20 µg), Student’s t-test was used. A red color * indicates PBS versus other and green color * indicates EV-DM versus EV-M and MAC-EVs. (**C**) Phase contrast microscopy images of migrated cells in 12 and 24 h, scale bar: 100 µm. (**D**) Quantified data of migrated cells shown in (**C**), Student’s t-test was used. * indicates PBS versus others; # indicates EV-DM versus EV-M and MAC-EVs. *p < 0.05; ** or ^##^*p* < 0.01; *** or ^###^*p* < 0.001. EV-M; media containing EVs; EV-DM; media containing no EVs.

MAC-EVs’ interaction with the DP cells is crucial for the activation of Wnt/β-catenin signaling via activation of receptors. To explore the cellular interaction of MAC-EVs, the DP cells were incubated with DiD-labeled EVs and confocal imaging was performed. The images showed that EVs predominantly accumulated in the membrane and internalization (arrow) of the DP cells. Furthermore, some of EVs were also internalized inside the DP cells (Figure 3A). These results suggested that MAC-EVs first interact with cell membrane before internalization.

### 3.4. MAC-EV Treatment Increases Cell Proliferation and Migration of DP Cells

The DP cells are especially important for hair growth; hence, we examined the effects of the EV-DM (100%), EV-M (100%), and MAC-EVs on DP cell proliferation and migration. Proliferation of DP cells increased significantly with EV-M (*p* < 0.05) and all the concentrations of MAC-EVs (*p* < 0.05 and *p* < 0.001) in a dose-dependent manner, compared to the PBS-control (Figure 3B). Proliferation of DP cells increased significantly with EV-M (*p* < 0.01) and all the concentrations of MAC-EVs (*p* < 0.01 and *p* < 0.001) in a dose-dependent manner, compared to the EV-DM (Figure 3B). No significant changes were observed between PBS and treatment with EV-DM. Significant increases were observed in DP cell migration by EV-DM, EV-M, and MAC-EVs compared to that with PBS (*p* < 0.05, *p* < 0.01, and *p* < 0.001) at 12 h. Similarly, the significant increases were observed in DP cell migration by MAC-EVs compared to that with EV-DM (*p* < 0.001) at 12 h. At 24 h, the significant increases were observed in DP cell migration by EV-M and MAC-EVs compared to that with PBS (*p* < 0.05, *p* < 0.01, and *p* < 0.001). Similarly, the significant increases were observed in DP cell migration by MAC-EVs compared to that with EV-DM (*p* < 0.01 and *p* < 0.001). At 12 h, a significant decrease was observed between PBS and EV-DM treatment (Figure 3C,D) and no significant difference was observed at 24 h. Treatment with MAC-EVs increased the migration and proliferation of DP cells, whereas EV-DM did not have any or longer such influence. These data suggest that both proliferation and migration were predominately specific to MAC-EVs.

### 3.5. MAC-EVs Increase the Levels of Marker Proteins, Survival- and Proliferation-Markers and Activate the Wnt/β-Catenin Signaling Pathway in DP Cells

**Figure 4:**
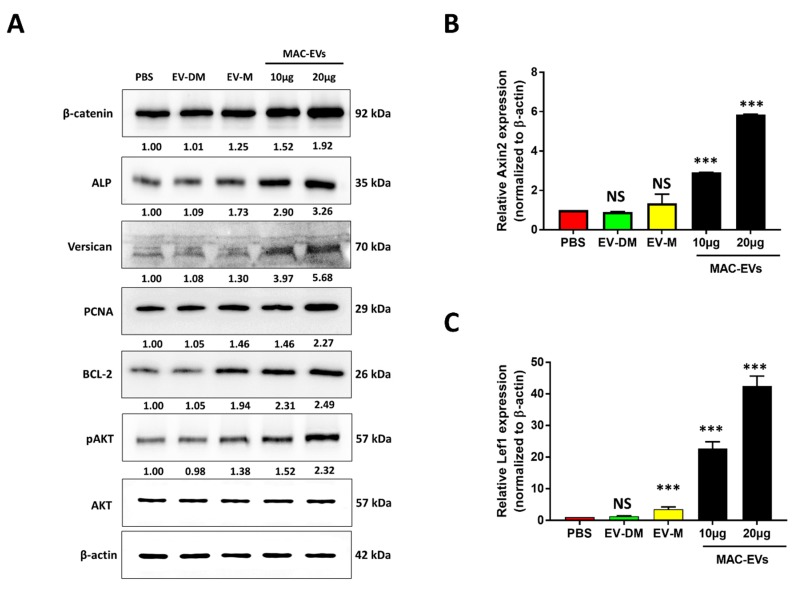
MAC-EV treatment leads to increased levels of hair-inductive proteins and survival and proliferation markers and activates the Wnt/β-catenin signaling pathway. (**A**) Western blot analysis of β-catenin, Versican and ALP, pAkt, Akt, Bcl-2, PCNA, and β-actin levels in DP cells after 24 h treatment of MAC-EVs. (**B**,**C**) qPCR results of mRNA expressions of *Axin2 and LEF1* in DP cells treated with EV-DM, EV-M, and MAC-EVs for 24 h (*n* = 3). Means ± SD of triplicate experiments are shown. ***p < 0.001. Student’s t-test was used for comparison. EV-M; media containing EVs; EV-DM; media containing no EVs.

To determine the effect of MAC-EVs on the hair-inductive activity of DP cells, we treated the DP cells with PBS, EV-DM, EV-M, and MAC-EVs, and evaluated the expression of proteins associated with anagen induction, such as β-catenin [30], Versican [31], and alkaline phosphatase (ALP) [32] (Figure 4A). The β-catenin, Versican, and ALP were expressed in DP cells at higher levels with MAC-EVs (10, 20 µg) treatment in a dose-dependent manner, compared to that in the control (Figure 4A). Furthermore, no changes were seen upon treatment with EV-DM when compared to the PBS-control; however, treatment with EV-M showed a substantial increase in the protein level. Further, we tested the activation the AKT signaling pathway and cell survival marker Bcl-2 and cellular proliferation marker PCNA (proliferating cell nuclear antigen). The MAC-EV treatment upregulated levels of phosphorylated AKT (pAKT) when compared to the control (Figure 4A). Furthermore, western blot results showed that Bcl-2 and PCNA were increased with MAC-EV and EV-M treatments; however, no changes were observed upon EV-DM treatment (Figure 4A).

Next, we examined the activation of Wnt/β-catenin target genes (*Axin2 and lef1*). Real-time PCR results showed that activation of β-catenin by treatment with MAC-EVs upregulated the mRNA expression of *Axin2* by 2-fold (*p* < 0.001) and 6-fold (*p* < 0.001) more than that in the control, when incubated with 10 and 20 µg MAC-EVs, respectively. No significant change was observed in treatments with EV-DM and EV-M as compared to PBS (Figure 4B). Similarly, mRNA levels of *lef1* also elevated by 4-fold (*p* < 0.001), 20-fold (*p* < 0.001), and 40-fold (*p* < 0.001) for incubation with EV-M and 10 and 20 µg of MAC-EVs, respectively (Figure 4C).

### 3.6. Treatment with MAC-EV Upregulates Expression of Hair Inducing Growth Factors in DP Cells

The mRNA expression of vascular endothelial growth factor (VEGF) and keratinocyte growth factor (KGF) was increased by EV-M and MAC-EVs treatment in a dose-dependent manner. In DP cells, treatment with EV-M increased levels of VEGF and KGF substantially compared to the control. Treatment with MAC-EV increased VEGF levels by 2.5-fold in DP cells compared to the control. KGF was increased 2.5- and 3.2-fold respectively, by the treatment compared to the control (Supplementary Figure S3). No changes were observed in EV-DM treatment compared to PBS. These results suggest that MAC-EV treatment could increase the expression of the growth factor genes in DP cells.

### 3.7. Determination of MAC-EVs Treatment Intervals in Balb/c Mice

**Figure 5:**
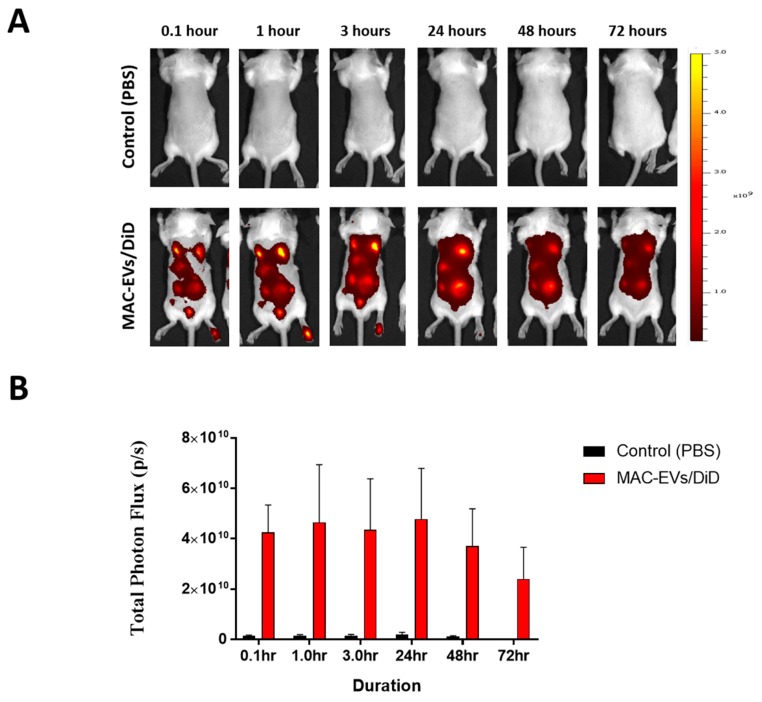
Determination of MAC-EV treatment intervals in Balb/c mice. (**A**) Time-based in vivo fluorescent imaging of MAC-EVs/DiD in Balb/c mice. MAC-EVs/DiD (*n* = 3) or PBS (control) (*n* = 3) was administered intradermally 2 days after hair was clipped. (**B**) Quantification of fluorescent signals from mice injected with MAC-EVs/DiD or PBS (control), Means ± SD of experiment are shown.

We calculated the retention time of MAC-EVs in Balb/c mice using DiD-labeled MAC-EVs (MAC-EVs/DiD) and in vivo fluorescence imaging. Mice whose dorsal hair had been removed were injected intradermally with MAC-EVs/DiD or Control (PBS), under the dorsal skin in multiple regions, and imaged at numerous time points: 0.1, 1, 3, 24, 48, and 72 h. Strong fluorescent signals were observed until 24 h on the dorsal sides of mice, which reduced after 48 h, and was less than half by 72 h. This suggests that, the MAC-EVs were retained in the dorsal skin of the mice up to 72 h (Figure 5A,B).

### 3.8. Hair Growth Effects of MAC-EVs in Balb/c Mice

**Figure 6:**
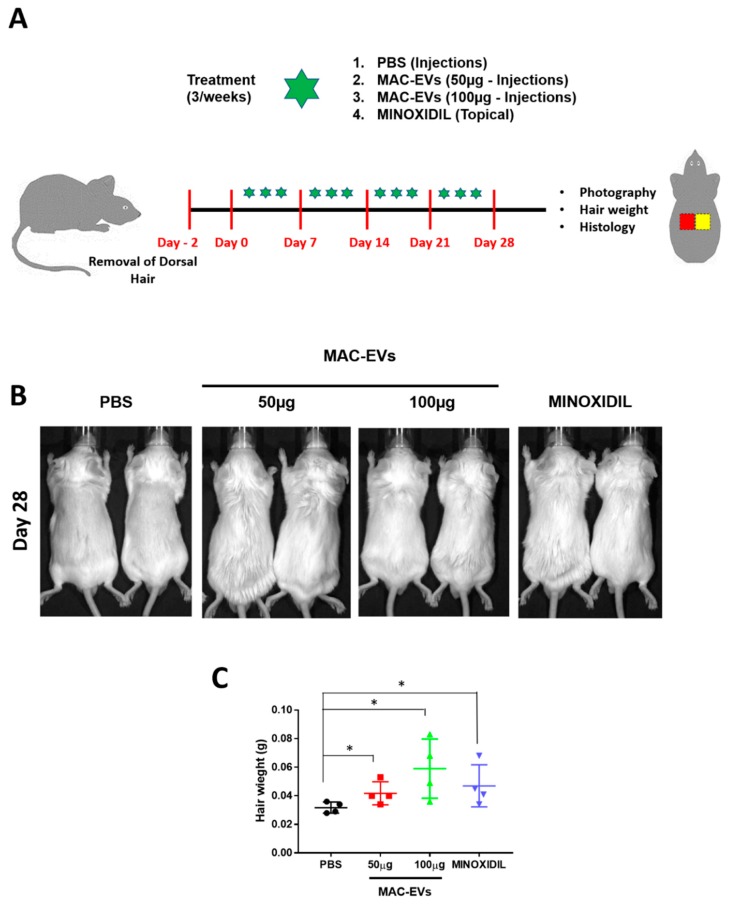
MAC-EVs induce growth of hair follicles in Balb/c mice. (**A**) Schematic of in vivo hair growth experiment with the mice (*n* = 7/group). (**B**) Representative images of dorsal skin at 28 day. (**C**) Weight of hair per cm^2^ area of dorsal skin (*n* = 4) at different treatment after 28 days (area of skin used for (**C**)) red color square represent with hair and yellow represent without hair (shaved before collection). Means ± SD of experiment are shown. *p < 0.05. Student’s t-test was used.

The hair growth ability of MAC-EVs was determined in Balb/c mice. We clipped the dorsal hair of Balb/c 2 days prior to the treatment. We compared the in vivo results of hair growth with MAC-EV treatments (50 and 100 µg), PBS-control group, and positive control of 3% minoxidil, which is considered as the current gold standard for hair growth treatment (Figure 6A). On day 28, we could observe an increased hair growth in the mice in the group treated with MAC-EV (50, 100 µg) and minoxidil than control groups. These results confirm that MAC-EV treatment can induce the hair growth in a mouse model (Figure 6B). The weight of newly grown hair in all groups was measured. It was found that weight of hair in MAC-EVs (100 µg)-treated mice was highest (*p* > 0.05) as compared to PBS-control mice. The weight of mice hair was approximately similar in MAC-EVs (50 µg) and minoxidil groups, however, significantly higher than the PBS-control group (*p* > 0.05; Figure 6C).

### 3.9. MAC-EVs Promote the HF Number and Dermis Thickness in Mice

**Figure 7:**
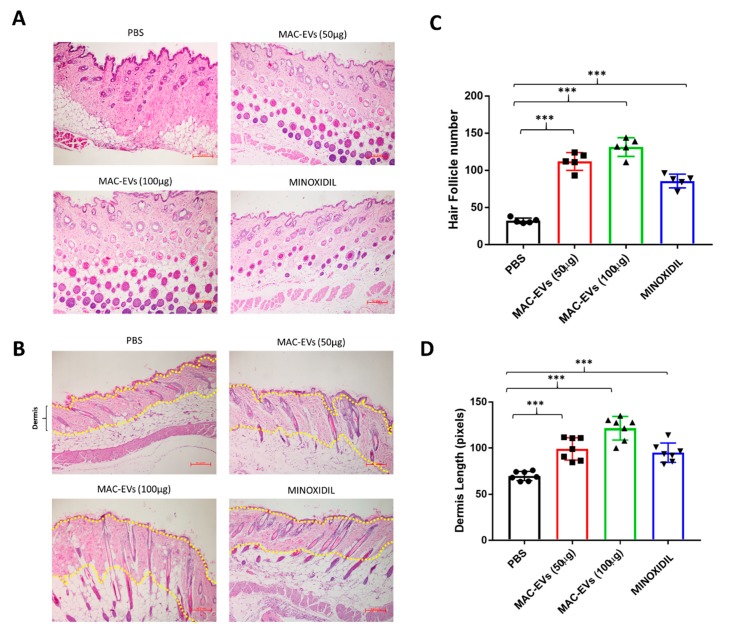
MAC-EVs promote the HF number and dermis thickness in Balb/c mice. (**A**) Transverse section of mouse dorsal skin after 28 days of treatment. (**B**) Cross-section of mice dorsal skin after 28 days of treatment (yellow dotted line indicates dermis). (**C**,**D**) Quantified data showing the number of hair follicles; absolute number per image field (*n* = 5), and dermis length (*n* = 8) in all groups. Means ± SD of at least triplicate experiments are shown. ***p < 0.001. Student’s t-test was used.

The effect of MAC-EVs on hair growth was further assessed by H&E staining. The treatment with MAC-EVs significantly (*p* < 0.001) increased the HF number in a dose-dependent manner compared to control. Similarly, minoxidil treatment also significantly (*p* < 0.001) increased the HF number as compared to the control, but this increase was lower than that with MAC-EV (100 µg) treatment (Figure 7A,C). MAC-EV and minoxidil treatments significantly increased thickness of dermis (*p* < 0.001) compared to the control group, but the minoxidil treatment increase in dermis thickness was lower than that with MAC-EV (100 µg) treatment (Figure 7B,D).

### 3.10. MAC-EVs Elongates the Hair Shaft of Human HFs

**Figure 8:**
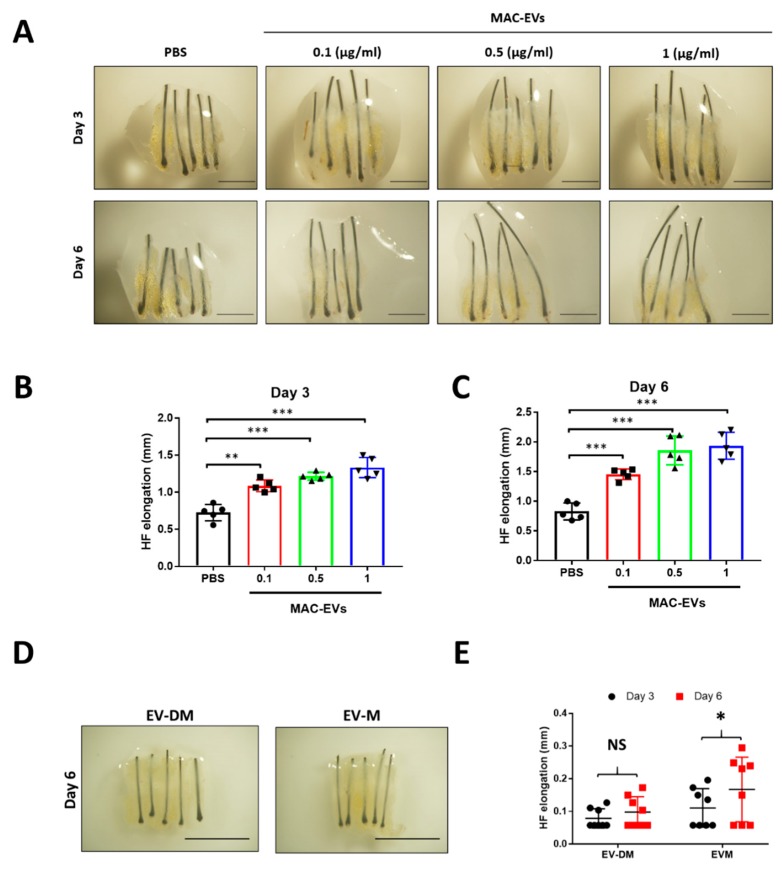
MAC-EVs promote hair follicle shaft elongation in human hair follicles. (**A**) Representative photographs of human hair follicles after PBS and MAC-EV treatment (0.1, 0.5, 1 µg/mL) (scale bar: 1 mm). (**B**,**C**) Quantified data of hair shaft elongation on day 3 and day 6 (*n* = 5). (**D**) representative photographs of human hair follicles after EV-DM and EV-M treatment (0.1, 0.5, 1 (µg/mL) (scale bar: 1 mm). (**E**) Quantified data of hair shaft elongation on day 3 and day 6 (*n* = 8). Means ± SD of experiments are shown. *p < 0.05; **p < 0.01; *** *p* < 0.001 Student’s t-test was used. EV-M; media containing EVs; EV-DM; media containing no EVs.

To determine the elongation of hair shaft, an organ culture experiment was performed with androgenic alopecia human scalp HFs treated with EV-DM, EV-M, and MAC-EVs (0.1, 0.5, and 1 µg/mL) and PBS, incubated for 3 and 6 days. The MAC-EV treatment increased the hair growth in individual #1 (Figure 8A). The day 3 results showed a significant increase (0.1 µg/mL MAC-EV versus PBS, *p* < 0.01; 0.5 and 1 µg/mL MAC-EV versus PBS, *p* < 0.001;) in elongation of hair shaft by the treatment in a dose- and time-dependent manner (Figure 8A,B). Likewise, the results of day 6 showed a significant (0.1 or 0.5 or 1 µg/mL MAC-EV versus PBS, *p* < 0.001;) elongation of hair shaft by the treatment in a dose- and time-dependent manner, no changes were observed in the control (Figure 8A,C).

Furthermore, MAC-EVs were tested in four more human subject hair follicles. In individuals #2 and #3, MAC-EVs treatment significantly (*p* < 0.01 and *p* < 0.001) increased the hair shaft growth in a time- and dose-dependent manner (Supplementary Figure S5). In individuals #4 and #5, MAC-EVs treatment significantly in 1 µg/mL (*p* < 0.05) and 0.5 µg/mL (*p* < 0.001) increased the hair shaft elongation, respectively. The substantial increase of hair shaft elongation was seen in a time-dependent manner (Supplementary Figure S4).

In individual #3, the EV-DM treatment did not significantly (*p* > 0.05) increase the length of hair shaft, on both day 3 and 6. The EV-M treatment significantly (*p* < 0.05) increased the length of hair shaft on day 6 (Figure 8D,E). The EV-DM and EV-M were tested in two more human subjects’ (individuals #4 and #5) hair follicles, and similarly significant (*p* *<* 0.05) results were obtained (Supplementary Figure S5).

## 4. Discussion

The DP cells located in the papilla of normal human HFs play a crucial role in the dermal–epidermal interactions that control hair production and events of the hair growth cycle. DP cells manage HF cycling through interacting between stem cells, endothelial cells, and HF stem cells [33,34]. A defect in any one of the cells can lead to hair loss. Thus, restoration of these cells for better interaction is considered a potential therapeutic strategy for treating hair loss. Results of the current study demonstrate that macrophage-derived EVs possess an ability to induce activation of DP cells in vitro. Furthermore, we also showed that MAC-EVs sufficiently enhance hair growth in a hair-clipped Balb/c mouse model by increasing the number of HFs and dermis thickness. 

In the current study, we have successfully isolated EVs from the supernatant of macrophage culture media and found their size and shape to be in agreement with the earlier reports [17,35]. MAC-EVs expressed the EV-specific biomarker markers, Alix, and cell organelle protein markers such as cytochrome-c, GM130, and calnexin, were not detected in EVs, confirming the isolation of pure EVs [6,14].

The Wnt proteins act as morphogens during development and can mediate the cell–cell communication over a large distance [36,37]. Several Wnt family members are expressed in distinct patterns and stages in the skin development of mammals and birds, such as Wnt1a, Wnt3, Wnt3a, Wnt4, Wnt5a, Wnt7a, Wnt10a, and Wnt10b [1,38]. Wnt1a, Wnt3a, Wnt7a, and Wnt7b are inducers of hair growth because of their ability to activate the Wnt/β-catenin signaling in DP cells [1,27,29,39]. The EVs function as mediators of cell–cell communication by selectively carrying biologically active molecules in the form of proteins and nucleic acids [6,40]. A study suggested that Wnts are secreted on exosomes both during drosophila development and in human cells. They also showed that exosomes carry Wnt3a on their surface to induce Wnt signaling activity in the target cells [41]. Owing to their membranous nature, exosomes are natural carriers for membrane-associated signaling proteins such as Wnts and Hedgehogs [42]. We evaluated whether the Wnt3a and Wnt7b proteins are present in macrophages or not and our results suggested their presence. Western blotting results further confirmed the presence of a more enriched amount of Wnt3a and Wnt7b in MAC-EVs than macrophages. As mentioned above, presence of Wnts in the membrane is necessary for activation of DP cell receptors, such as Frizzled and LRP5/6, and the Wnt3a and Wnt7b present in the MAC-EVs are mostly (more than 95%) associated with EV membrane. These results collectively demonstrated that MAC-EVs contain enriched amounts of Wnts, associated with the membrane, which could readily activate the Wnt/β-catenin signaling pathway in DP cells on interaction with their membrane. A recent study showed that activated fibroblast EVs delivered the Frizzled4 receptors to DP cells and activated the Wnt/β-catenin signaling pathway in DP cells [43].

Cytokines and growth factors that are secreted by a cell into the extracellular space bind to their specific receptors present on the surfaces of nearby cells. However, the discovery of EVs signifies an exciting scientific niche that suggests novel mechanisms by which paracrine signaling can be accomplished. EV interactions with the plasma membrane and its internalization are important for the activation of receptors and delivery of the biological materials into the recipient cells [44,45]. We confirmed the MAC-EV interaction with DP cells, subsequent internalization using labelled MAC-EVs with fluorescence. This process readily occurred within an hour of the treatment, in a dose-dependent manner [6].

A study compared the cultured DP cells from balding and non-balding scalp and found that balding DP cells showed characteristics of senescence, including loss of proliferation [46]. Cultured DP cells lose their ability of inducing hair follicle after sub-culturing in vitro [47], showing that DP cell proliferation is essential for the morphogenesis and growth of the HF [2]. In the current study, MAC-EVs were shown to have the capacity to induce proliferation of DP cells, which was further confirmed by upregulated PCNA expression. This result is in line with a study showing the activated fibroblast-derived EVs could induce cell proliferation signaling in DP cells [43]. This could be very useful for large-scale production of DP cells in vitro for research studies and DP cells therapies [47,48], as cultured DP cells lose their ability of inducing and maintaining the HF. This also could maintain the ability of proliferation of DP cells in vivo. MAC-EVs accelerate the motility of the DP cells in vitro and a previous study suggests that during the normal hair cycle, dermal sheath cup cells may also be a source of DP cells, via migration [49]. These results support the findings of our previous data on MSC-EVs [2].

The canonical Wnt signaling pathway leads to stabilization and accumulation of β-catenin, resulting in nuclear translocation and activation of *Axin2* and *Lef1*, the target genes of the Wnt/β-catenin pathway [28,50,51,52]. Results of the current study showed that treatment with MAC-EVs upregulated the β-catenin level in the human DP cells in a dose-dependent manner. This confirmed that the Wnt ligand, Frizzled and LRP5/6 receptor interaction in DP cells, upregulates the β-catenin level. Additionally, qPCR results of upregulated mRNA levels of transcription factors (*Axin2* and *Lef1*) further confirmed the function of translocated β-catenin in DP cells, by activating transcription factors important for anagen induction and maintenance. Our results are in agreement with a recent study, which showed that macrophages present near the hair follicles are positive for Wnt proteins, and also showed secreted Wnt10a near the DP cell and activates *axin2* and *Lef1* [21]. This study proved that Wnt3a/Wnt7b associated with MAC-EVs induced hair growth by activating receptors and relayed β-catenin cascade in DP cells. If β-catenin is unavailable, then the keratinocytes fail to form, which leads to failure in the development of hair [53]. Recently, a study suggested that aspartate-serine-serine peptides promote hair growth through the activation of β-catenin signaling and the expression of nuclear β-catenin and phosphorylation of AKT, both of which are key factors of the β-catenin pathway in DP cells [54]. Another study showed that minoxidil translocated β-catenin into the nucleus [55]. Both studies showed either anagen prolongation or telogen to anagen transition, and our results were consistent with the results of these studies. Although the relationship between Wnt3a/Wnt7b is shown in EVs and β-catenin activation in DP cells, a direct demonstration of their involvement requires further investigation. The treatment of DP cells with MAC-EVs upregulated the expression of other hair-inducing marker proteins, such as Versican [31] and ALP [32], as measured by western blotting. Our results are consistent with these findings and the necessity of hair-inducing activity in DP cells for hair growth [5,56,57]. A previous study reported that Wnt3a has a significant ability to restore the ALP activity of DP cells [58]; similarly, we observed that MAC-EV treatment increased the ALP in DP cells. Survival of DP cells is essential for HF development and function. The DP cells are also a reservoir with the potential to differentiate into a range of cells that have potential therapeutic importance in hair growth [56]. Our results suggest that MAC-EVs lead to activation of Akt phosphorylation and increase Bcl-2 (anti-apoptotic regulator) expression in DP cells. Survival rate of cells is mainly determined by Akt and Bcl-2, which play a critical role in mediating survival signals [59,60].

DP cells are capable of releasing growth factors that direct epithelial cells to proliferate, leading to hair shaft growth and acceleration of hair growth [2,33]. The nuclear translocation of β-catenin leads to activation of *Axin2* and *TCF/LEF*, the Wnt/β-catenin pathway target genes [50,61]. This also leads to activation of the VEGF gene [62]. Our results revealed that VEGF gene expression and release were significantly increased in a dose-dependent manner by MAC-EV treatment. Several studies have shown that VEGF and KGF can promote hair growth [33,63,64,65,66], and MAC-EV treatment also increased the expression of KGF. These secretory cytokines may further induce HF interaction with other cells (such as outer root sheath cells). All the in vitro EV-DM were shown to affect/influence the results when compared to PBS control, but EV-M minimally induced DP function. Thus, this confirms that induction/activation of DP cells and their function is mainly specific to MAC-EVs. We showed that Wnt3a and Wnt7b involvement in activation of the Wnt/β-catenin signaling pathway and induction of hair growth ability of DP cells are essential. We do not rule out contribution from other proteins or miRNAs in MAC-EV alone or in combination, to the hair inducting activity. Moreover, proteomics and miRNA microarray analysis of MAC-EV may provide a complete picture of MAC-EV-mediated activity.

Balb/c mice were used to evaluate the in vivo hair growth effects by MAC-EV treatment. Before the hair growth in vivo study, we showed the MAC-EVs retention time in mice skin by live animal imaging, and it showed that MAC-EVs stayed more than 72 h and spread to other parts of dorsal skin of mice. So, the treatment interval was determined as 72 h. Hair clipping or plucking were used in the hair growth studies [2,67,68]. We used a most commonly used method of clipping (shaving the hair) for our study. A report suggests that plucking of hair grows faster than clipping in guinea pig [69]. We selected the clipping model for our study to show the real effect of MAC-EV treatment, which is not affected by the model (plucking). We demonstrated that MAC-EV treatment induces faster hair growth compared to minoxidil treatment, the current gold standard. In our previous study, MSC-EVs showed an effect comparable to minoxidil [2]. We also measured the weight of hair/cm^2^ area of dorsal skin, and our results suggested that MAC-EV treatment improves the hair growth faster than minoxidil and the control. Further histological results confirmed that MAC-EV treatment increases the number, which confirms the hair growth effect of MAC-EVs and induction of de novo HFs. Low-dose MAC-EV treatment significantly promoted dermis thickness, comparably to the minoxidil group, and high-dose MAC-EV treatment showed more enhanced dermis thickness. Thickness of dermis, which indirectly reflects enhancement of telogen to anagen transition [2,70,71], was also increased by MAC-EV treatment. Minoxidil treatment causes irritation and allergic reaction on the scalp in some patients [72], and visible hair growth diminishes after discontinuation of the treatment [73]. Long-term effects of MAC-EVs treatment on hair growth remain to be investigated.

Since we could not test the effect of MAC-EVs in humans, we used hair follicles isolated from humans and tested them in in vitro conditions for the effect on hair shaft elongation using an organ culture of human scalp HFs. This experiment is widely used in preclinical studies [28,47,74], before testing in clinical trials. Our results suggested that MAC-EVs could elongate the human hair shaft by two-fold within 6 days. Hair shaft growth was not induced by EV-DM, but EV-M showed significant growth.

As mentioned earlier, for patients with hair loss, drugs such as finasteride and minoxidil are clinically approved by US Food and Drug Administration. However, minoxidil is known for its activation of the β-catenin pathway in human DP cells, but hair growth was observed to be minimal or stopped after discontinuation of the drugs [2,5,55,73]. Thus, there is a need for new drugs or alternative therapeutic materials. The EVs derived from macrophage could be excellent candidates for stimulating hair growth in humans, since their isolation from the same patients is relatively easy and less invasive than isolation of mesenchymal stem cells from the adipose tissue or bone marrow.

## 5. Conclusions

We showed the presence of Wnt3a/Wnt7b proteins in MAC-EVs, which are associated with the membrane. We also demonstrated that MAC-EVs promoted proliferation and migration and the activation of the Wnt/β-catenin signaling pathway in DP cells. The MAC-EVs enhanced the hair-inductive properties of DP cells by increasing the levels of hair-inductive proteins and survival/proliferation markers in vitro. Further experiments revealed that MAC-EVs promote hair growth through stimulation of VEGF and KGF in DP cells. In vivo results showed that MAC-EVs accelerate hair growth by increasing the number of HFs and dermis thickness. Finally, we showed the human HF shaft elongation by MAC-EV treatment. These findings represent an advancement within the field of HF growth by EVs. 

## Supplementary Materials

The following are available online at https://www.mdpi.com/2073-4409/9/4/856/s1. Supplementary Figure S1. Immunofluorescence analysis of CD68 in murine macrophage cell; Supplementary Figure S2: Schematic diagram of the generation and purification of MAC-EVs; Supplementary Figure S3. Effects of MAC-EVs expression of growth factors in DP cells; Supplementary Figure S4. MAC-EVs promotes the hair follicle shaft elongation in human; Supplementary Figure S5. EV-M promotes the shaft elongation in human hair follicles; Table S1: List of antibodies used in this study; Table S2: RT-PCR primers list; Table S3: Quantitative PCR primers list.
