# Supplementary material for: Macrophage-Derived Extracellular Vesicle Promotes Hair Growth
Source: Cells. 2020 Apr 1;9(4):856. doi: 10.3390/cells9040856 (PMC7226775; doi:10.3390/cells9040856)
Supplement: Supplementary file 1 [file cells-09-00856-s001.pdf]

Macrophage-Derived Extracellular Vesicle Promotes Hair Growth

Ramya Lakshmi Rajendran [1,2,†], Prakash Gangadaran [1,2,3,†], Chang Hoon Seo [4], Mi Hee Kwack [5], Ji Min Oh [1,2,3], Ho Won Lee [1,2], Arunnehru Gopal [1,2], Young Kwan Sung [5], Shin Young Jeong [1,2], Sang-Woo Lee [1,2], Jaetae Lee [1,2] and Byeong-Cheol Ahn [1,2,3,*]

[1] Department of Nuclear Medicine, School of Medicine, Kyungpook National University, Daegu, Republic of Korea.

[2] Department of Nuclear Medicine, Kyungpook National University Hospital, Daegu, Republic of Korea.

[3] BK21 Plus KNU Biomedical Convergence Program, Department of Biomedical Science, School of Medicine, Kyungpook National University, Daegu, Republic of Korea.

[4] New Drug Development Center, Daegu-Gyeongbuk Medical Innovation Foundation (DGMIF), Daegu, Republic of Korea.

[5] Department of Immunology, School of Medicine, Kyungpook National University, Daegu, Republic of Korea.

[†] These authors contributed equally to this study

***Correspondence:** abc2000@knu.ac.kr

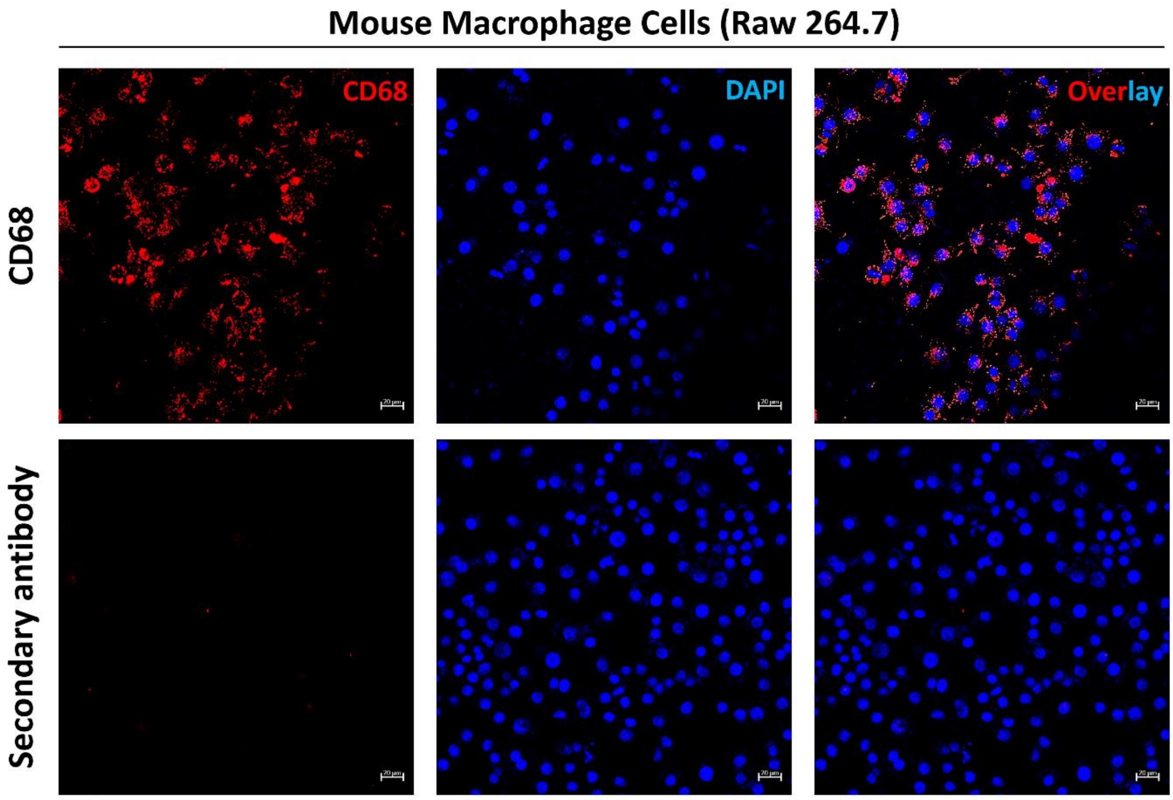

Figure S1. Immunofluorescence analysis of CD68 in murine macrophage cell (Raw 264.7) (scale bar, 20 μm).

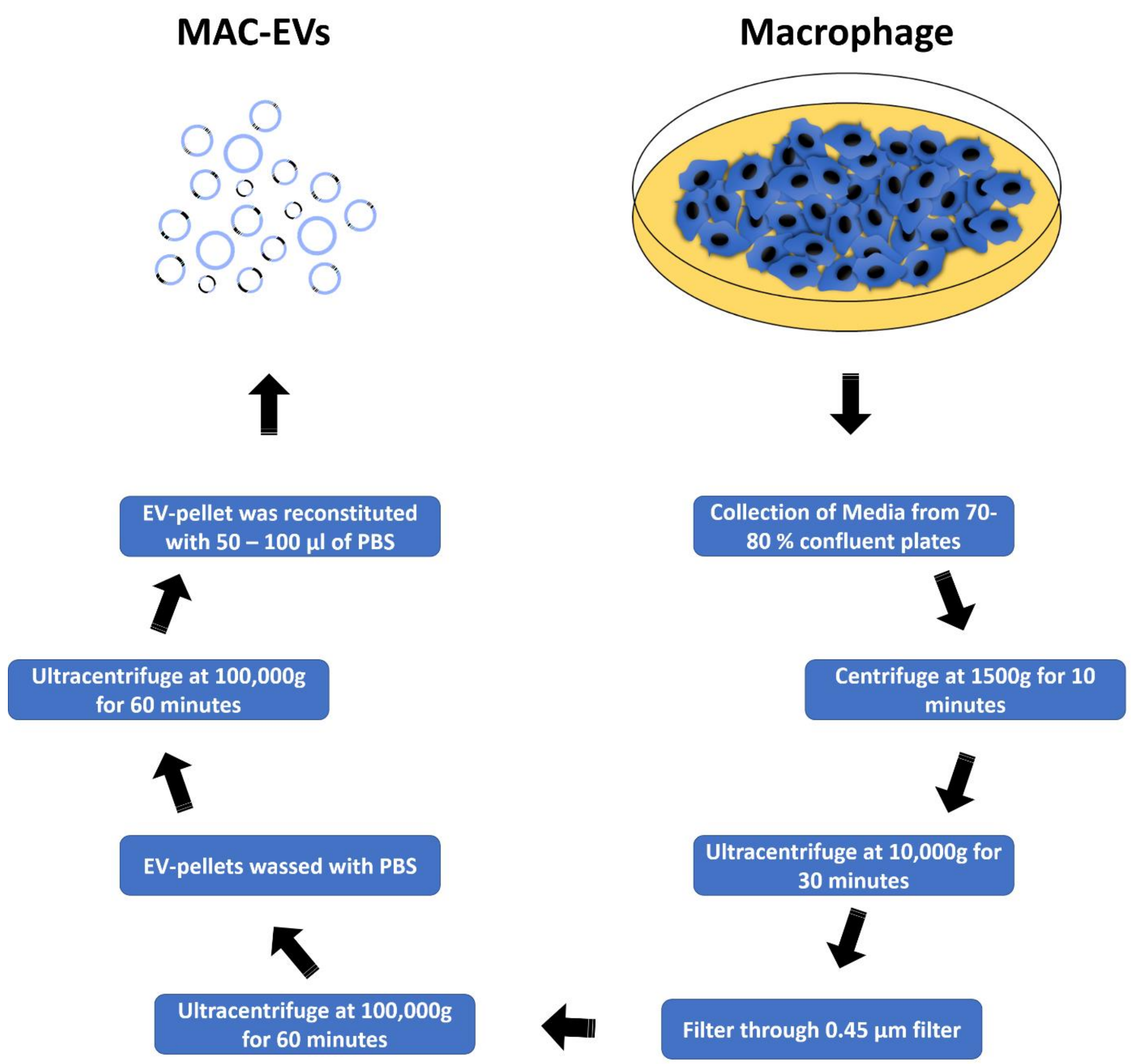

Figure S2. Schematic diagram of the generation and purification of MAC-EVs.

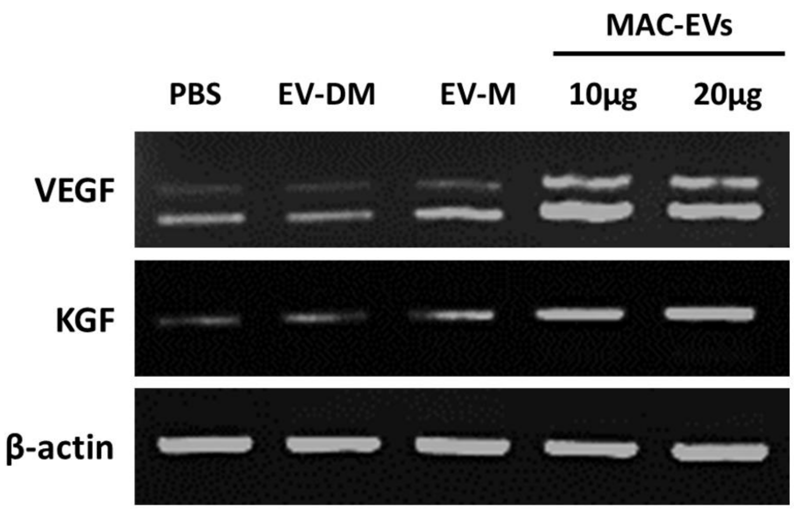

Figure S3. Effects of MAC-EVs expression of growth factors in DP cells. Gel image of reverse transcription polymerase chain reaction (RT-PCR) gene expression of DP cells treated with MAC-EVs for 24 h.

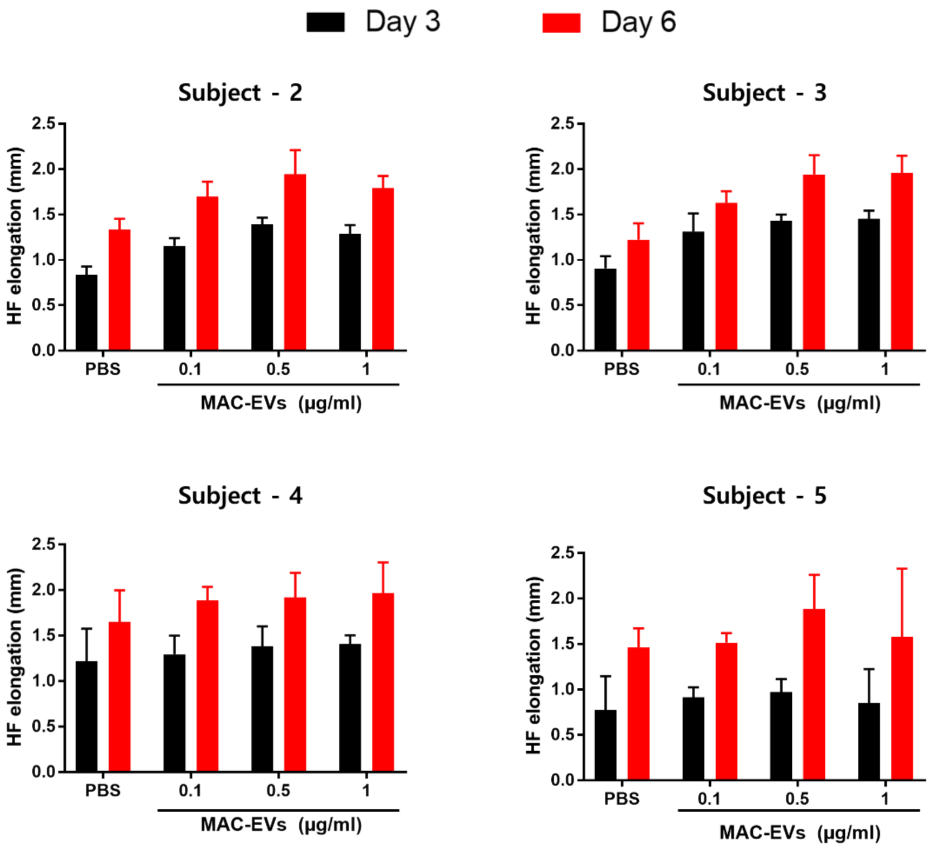

Figure S4. MAC-EVs promotes the hair follicle shaft elongation in human. Quantified data of hair shaft elongation (n = 5–8) in day 3 and day 6. Mean ± SD of experiments are shown. Two-way ANOVA was used.

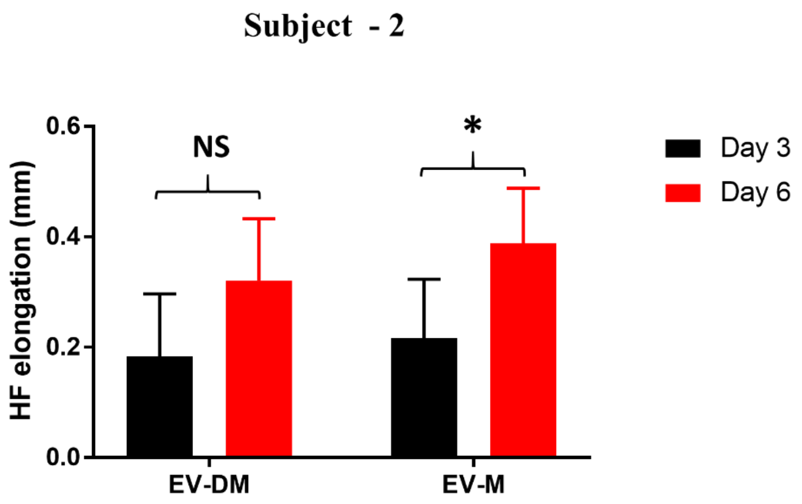

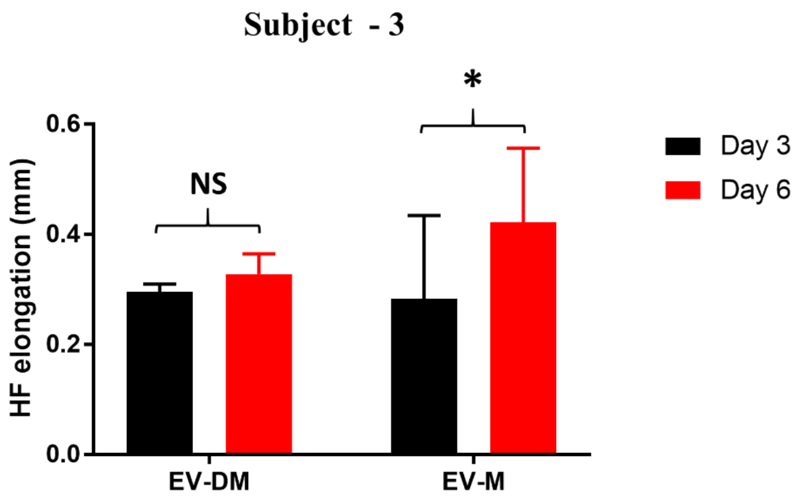

Figure S5. EV-M promotes the shaft elongation in human hair follicles. Quantified data of hair shaft elongation (n = 5–8) in day 3 and day 6. Mean ± SD of experiments are shown. *Student-t-test* was used. EV-DM: EV depleted Media, EV-M: media contains EVs**.**

Table1. List of antibodies used in this study.

Antibody	Company	Dilution	
		Western blotting	Immunofluorescence
ALIX	Abcam	1:5000	
CD63	Abcam	1:5000	
TSG101	Abcam	1:8000	
GM130	Abcam	1:4000	
Calnexin	Abcam	1:4000	
Cytochrome C	Abcam	1:4000	
β-catenin	Cell signaling	1:4000	1: 500
Versican	Abcam	1:4000	1: 400
ALP	Abcam	1:4000	1: 400
pAKT	Cell signaling	1:5000	
AKT	Cell signaling	1:5000	
Bcl-2	Cell signaling	1:8000	
PCNA	Cell signaling	1:6000	
GAPDH	Cell signaling	1:10000	
β-actin	Cell signaling	1:6000	
Wnt3a	Abcam	1:4000	1 :200
Wnt7b	GeneTex	1:4000	1 :200
CD68	Abcam		1: 200
HRP conjugated anti-mouse	Cell signaling	1:10000	
HRP conjugated anti-rabbit	Cell signaling	1:10000	
Alexa Fluor 555 conjugated anti-rabbit	Cell signaling		1:200
Anti-goat rabbit -FITC	Cell signaling		1:400

Table 2. RT-PCR primers list.

**Gene**	Forward	Reverse
VEGF	GCGAGTCTGTGTTTTTGCAG	TCTTCAAGCCATCCTGCGTG
KGF	AATTCCAACTGCCACTGTCC	GACATGGATCCTGCCAACTT
β-actin	GGGAAATCGTGCGTGACATT	GGAGTTGAAGGTAGTTTCGT

Table 3. Quantitative PCR primers list.

Gene	Forward	Reverse
Axin2	AGCTAGGAGTGCGTTCATGGTT	GGAGGGACGTAGTGCAAAGC
LEF1	CAGGAGCCCTACCACGACAA	CCTCCATCTGGATGCTTTCC
β-actin	GGGAAATCGTGCGTGACATT	GGAGTTGAAGGTAGTTTCGT
